# Celiac disease diagnosis: transglutaminase, duodenal biopsy and genetic tests correlations

**DOI:** 10.3389/fped.2024.1330511

**Published:** 2024-08-29

**Authors:** Katia Regina Pena Schesquini-Roriz, Gloria Maria Fraga Rodríguez, Jocelyn Cristina Betancourt Castellanos, Laura Martinez-Martinez, Susana Boronat Guerrero, Carlos Rodrigo, Isabel Badell

**Affiliations:** ^1^ Department of Medicine, Federal University of Rondonia, Porto Velho, Brazil; ^2^ Department of Pediatrics, Hospital de la Santa Creu i Sant Pau, Autonomous University of Barcelona, Barcelona, Spain; ^3^ Department of Immunology, Hospital de la Santa Creu i Sant Pau, Universitat Autònoma de Barcelona, Barcelona, Spain; ^4^ Pediatric Service, Hospital Germans Trias i Pujol, Badalona, Spain

**Keywords:** celiac disease, diagnosis, HLA—DQ antigens, transglutaminase (TGA), duodenal biopsies

## Abstract

**Introduction:**

Celiac disease (CD) is an autoimmune enteropathy triggered by gluten ingestion in genetically susceptible individuals. The haplotypes HLA-DQ2 and DQ8, transglutaminase (TGA) antibodies, and biopsy findings are the main tests performed in the evaluation and CD diagnosis. The objective was to establish possible correlations between transglutaminase levels, genetic markers tests, and qualitative intestinal biopsy findings (modified Marsh classification) at the diagnosis.

**Methods:**

A retrospective cohort study. The selection criteria were confirmed CD cases with genetic tests performed. Statistical analysis was done mainly through One-way ANOVA, Kendall's correlation coefficient (T), and linear regression.

**Results:**

The study included 112 patients, with a mean age of 6 ± 4 years. All cases were tested to HLA-DQ2, and it was positive in 93%. HLA-DQ8 was tested in 73% of cases and it was positive in 61%. The percentage of negative genetic markers (DQ2/DQ8) was 4.5% for patients tested to both haplotypes. A comparison of DQ2/DQ8 (positive and negative) with clinical findings and tests performed did not identify any differences for most of the parameters analyzed. Cases of type I diabetes presented significant negative expression for DQ2(−); *p* = 0.05 and positive expression for DQ8(+); *p* = 0.023. The TGA antibody levels ranged from 18 to 36,745 U/ml. An inverse correlation was found between age and TGA-L level (*p* = 0.043). In 23% of the cases, the TGA levels were greater than 1,000 U/ml and presented a moderate positive correlation with the atrophy biopsy profile (*T* = 0.245). Patients with an atrophic biopsy profile (Marsh III) had a moderate positive correlation with growth failure (*T* = 0.218) but a negative correlation with constipation (*T* = −0.277).

**Conclusion:**

In terms of diagnosis tests for CD, transglutaminase levels and age presented an inverse correlation, with the level decreasing as age increased. A moderately positive correlation was found between mean transglutaminase with intestinal atrophy and growth retardation. The genetic test DQ2 was positive for 93% and negative genetic markers (DQ2/DQ8) represented 4.5% of cases studied.

## 1 Introduction

Celiac Disease (CD) is a multifactorial disorder with complex genetic predisposition. The general population prevalence is estimated at 1% (1). The genetic human leukocyte antigen haplotypes (DQ2/DQ8) are one of the most important predisposing genetic factors. Caucasian populations with these positive genetic markers have a higher prevalence for CD than the general population, with an estimated rate of 3% (2, 3). Around 90%–95% of the CD patients present these main haplotypes (4, 5). The presence of these genetic markers has an important role in the mechanism of this immune illness with systemic manifestations triggered by gluten consumption (6). Although, a varied percentage of CD cases cannot express these genetic markers which is estimated in less than 5% (7).

One of the main factors involved in these mechanisms is the function of transglutaminase. It has a crucial role in the autoimmunity mechanism against gluten proteins (8). According to literature, transglutaminase antibodies are the main diagnostic test with a positive predictive value greater than 95% (9, 10).

This study aimed to assess the correlations among transglutaminase levels, genetic markers (DQ2 and DQ8), and biopsy findings at diagnosis. The second objective was to determine the correlation between transglutaminase level and age.

## 2 Materials and methods

### 2.1 Study design

It is a study of confirmed cases of CD. The diagnosis was based on the criteria of the European Society of Pediatric Gastroenterology, Hepatology, and Nutrition (ESPGHAN) (9, 11). Data were collected retrospectively from 2008 to 2018. The manuscript building has followed the STROBE Statement for observational studies and the checklist of items for cohort study.

### 2.2 Patients

The sample size for this study corresponded to all cases from the pediatric service who underwent genetic tests performed at the time of the study. The selection criterion for this study was children (less than 18 years old) with confirmed cases who underwent genetic testing (HLA DQ2/DQ8). In the analysis, the genetic test for first-degree relatives (FDR) was considered when all family members (parents and siblings) were tested. The main parameters analyzed were demographic characteristics, clinical symptoms (according to the Oslo classification), duodenal biopsy, antibodies, and HLA-DQ tests.

### 2.3 Tests

All cases underwent total IgA measurements. IgA-deficiency cases were diagnosed using IgG class antibody tests (TGA and EMA). Serum IgA was measured using ELISA kit (Phadia™, Thermo Fisher Scientific) with normal levels according to the age range in years (1–4 > 15 mg/dl, 5–11 > 35 mg/dl and 12–15 > 40 mg/dl). The anti-endomysium antibody (EMA) IgA or IgG was determined by indirect immunofluorescence using the esophagus of a monkey as the substrate. It was expressed as a negative or positive test. The transglutaminase antibody IgA (TGA) was measured using an ELISA kit (Phadia™, Thermo Fisher Scientific) with a positive level ≥ 18 U/ml. In cases of two TGA measurements, a higher value was considered for the analysis.

HLA-DQ haplotypes were performed using a kit of polymerase chain reaction with DNA amplification for allele identification (Olerup SSP®). The kit detects the haloptypes DQ2 (DQ2.2, and DQ2.5) and DQ8 (4). They were classified as “positive” or “negative” results. A double test for both positive and negative genetic tests was routinely performed by immunogenetic laboratory. The intestinal biopsy was interpreted according to the modified Marsh-Oberhuber classification (12, 13). It will be referred in this article as Modified Marsh classification. The cases were subclassified into two groups: non-atrophic profile (grade 0, I, or II) and atrophic profile (grade IIIa, b, or c). Only the tests performed at the time of diagnosis were considered in the analysis.

### 2.4 Statistical methods

For quantitative variables, the mean or median was used depending on the normality of the distribution. Chi-square or Fisher's exact test was used for categorical data. Based on the study by Smarrazzo et al., a decimal logarithm base 10 transformation was performed for the TGA samples (TGA-L) to reach a normal distribution (14). TGA-L was used in the multivariate analysis through the Oslo classification, age at diagnosis, biopsy profiles, and genetic tests for patients. One-way ANOVA was performed for pairwise multiple comparisons means (TGA-L, age, biopsy subtypes). *Post hoc* tests (Bonferroni and Tukey tests) were performed to determine which means differed and are presented in graphics with asterisks indicating the significantly different group means at an alpha level of 0.05. Kendall rank correlation was performed to non-parametric variables (atrophic and non-atrophic profiles biopsy) due to the more assumptions for no small sample size. The Kendall's correlation coefficient (T) varies between +1 and −1. It was calculated based on clinical findings, genetic markers, and transglutaminase levels. For multivariate analysis, regarding the TGA levels (dependent variable) with age and biopsy subtypes (covariables), a linear regression was performed, and it is presented in graphic representation. A 2-tailed *p* < 0.05 was considered as having statistical significance. Statistical analyses were performed using the IBM SPSS version 27.0. (IBM Corp., Armonk, NY, USA).

## 3 Results

### 3.1 Baseline characteristics of the patients

In total, 112 children with a confirmed diagnosis of CD were included in this study. The mean age was 6 ± 4 years. Main characteristics of the patients are shown in Table 1.

**Table 1:** Main characteristics of the pediatric celiac disease cases studied.

Characteristics Total (*n* = 112)	Frequencies	Percentage
Gender		
Male	47	42%
Female	65	58%
Development periods		
<2 years of age—toddler	34	33%
2–12 years of age—child	55	53%
>12 years of age—teenager	15	14%
Onset of symptoms (before diagnosis)		
<2 years	73	66%
>2 years	39	34%
Family CD history (at least one member)		
Yes	17	15%
No	95	85%
Oslo Classification		
Classical	77	69%
Non-classical	35	31%
Autoimmune diseases		
Type 1 diabetes	6	5.4%
Hypothyroidism	4	3.6%
Dermatitis Herpetiformis		
Yes	3	3%
No	109	97%

### 3.2 First-degree relatives: genetic markers

**Figure 1:**
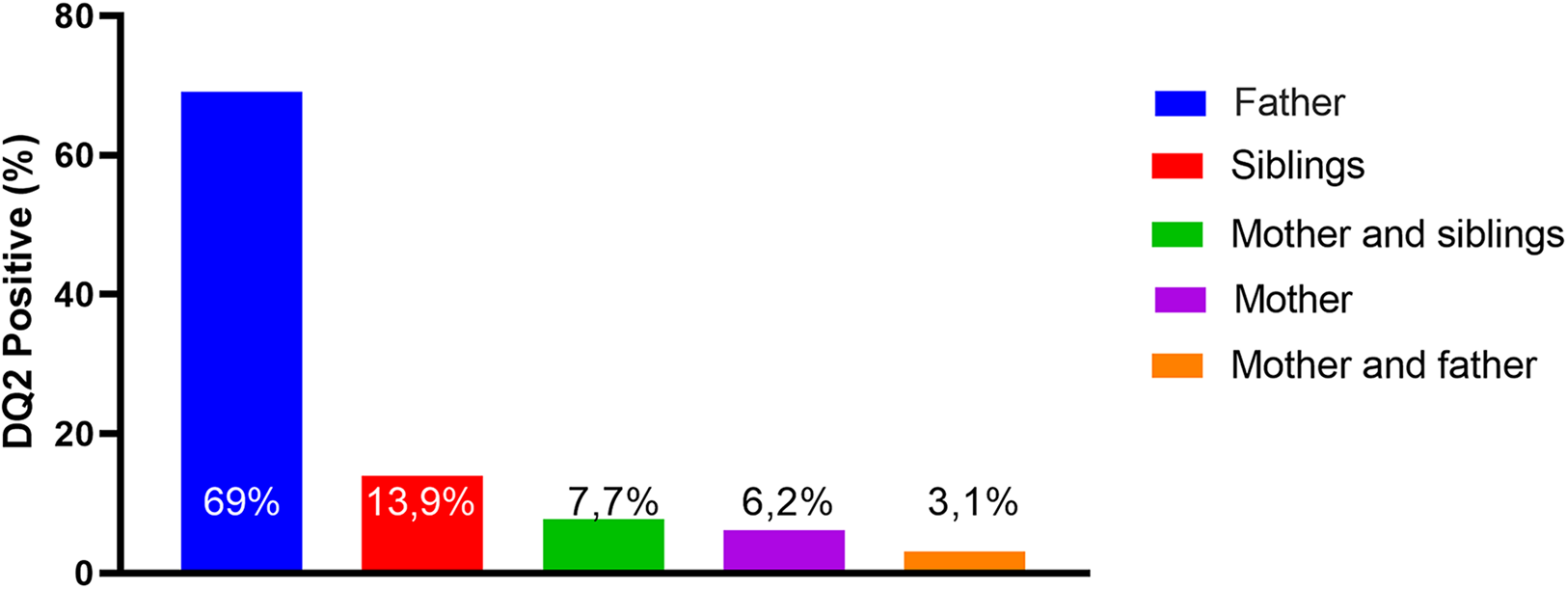
The expression of positive DQ2 among first-degree relatives (FDRs).

HLA-DQ2 was performed in 58% of First-degree relatives (FDRs) for all family members (parents and siblings). They were not tested for DQ8. DQ2 haplotype analysis was positive for one family member in 35% and for two members in 11%. Regarding the genetic expression per family member, the father was substantially the main positive member (69%) (Figure 1). As expected, patients with a family history of CD presented a significantly higher rate of DQ2 positivity than those without a family history (90% vs. 26%, respectively; *p* < 0.001).

### 3.3 Patients: genetic markers

All patients underwent at least one genetic test: DQ2(100%) and QD8 (73%). Positivity rates were 93% and 61%, respectively. Negative DQ2 and DQ8 were found in 4.5% of cases. Luckily, all those DQ2 negative have also taken the DQ8 test. In fact, this subgroup represents the cases of negative genetic markers for DQ2 and DQ8 in the population studied. The analysis of these groups did not show differences for all parameters analyzed (clinical findings, serological tests, and duodenal biopsy) in comparison with HLA-DQ2 or DQ8 positivity.

The selected cases were initially allocated and compared into two groups, DQ2(−) and DQ2(+) (Table 2). The DQ2(+) group was subdivided into two new groups (DQ8(+) and DQ8(−) (Table 3). The DQ2(+) and DQ2(−) groups did not show differences in most of the parameters analyzed. Only “type I diabetes” was significantly higher in the group DQ2(−) 25% than the DQ2(+) 4%; *p* = 0.05. The comparison of subgroups, DQ2(+)/(DQ8(+) and DQ2(+)/DQ8(−), did not reach statistical significance for most variables analyzed but “type I diabetes” was significantly higher in the subgroup DQ8(+) than DQ8(−), 13% vs. 0%; *p* = 0.023.

**Table 2:** Clinical findings and tests performed: DQ2(+) and DQ2(−) groups.

Characteristics/tests	DQ2 negative *N* = 8 (7%)	DQ2 positive *N* = 104 (93%)	*p-*value
Age (Y)	6 ± 4	7.8 ± 5	0.29
Female	9%	91%	0.46
Family history	25%	14%	0.34
Onset of symptoms > 2 years	37%	35%	0.57
Classical profile	50%	70%	0.25
Growth Failure	0%	26%	0.19
Diabetes	25%	4%	**0**.**05**
Hypothyroidism	0%	3.8%	1
Herpetiform Dermatitis	0%	2.9%	1
Other alimentary allergies	0%	8%	1
DQ2(+) rate for FDR	25%	36%	1
IgA Total mean (mg/dl)	132 ± 54	124 ± 73	0.75
TGA-L (mean ± SD)	2.2 ± 1	2.4 ± 0.7	0.66
EMA (+)	100%	78%	0.19

TGA-L, transglutaminase antibody IgA class in logarithm base; EMA, endomysial antibodies IgA class; FDR, first-degree relatives; DQ2, haplotypes HLA-DQ2; SD, standard deviation.

*p*-value with statistical significance in bold.

**Table 3:** Clinical findings and tests performed between the DQ2(+)/DQ8(+) and DQ2(+)/DQ8(-) groups.

Characteristics/tests	DQ2(+)/DQ8(+) *n* = 30 (40%)	DQ2(+)/DQ8(−) *n* = 45 (60%)	*p-*value
Age (Y)	6.9 ± 4	5,5 ± 4.3	0.19
Female	65%	35%	0.2
Family history	13%	9%	0.4
Onset of Symptoms: >2 years	46%	54%	0.4
Classical profile	37%	63%	0.47
Growth Failure	13.3%	28%	0.9
Diabetes	13.3%	0%	**0**.**023**
Hypothyroidism	6.7%	2.2%	0.35
Herpetiform Dermatitis	3.3%	2.2%	0.6
Other alimentary allergies	7%	7%	0.9
DQ2(+) rate for FDR	38%	23%	0.32
IgA Total (mg/dl)	124 ± 78	109 ± 78	0.42
TGA-L (mean ± SD)	2.4 ± 0.6	2.3 ± 0.7	0.6
EMA (+)	85%	78%	0.49

TGA-L, transglutaminase antibody IgA class in logarithm base; EMA, endomysial antibodies IgA class; FDR, first-degree relatives; DQ2, haplotypes HLA-DQ2; SD, standard deviation.

*p*-value with statistical significance in bold.

### 3.4 Diagnostic tests

**Figure 2:**
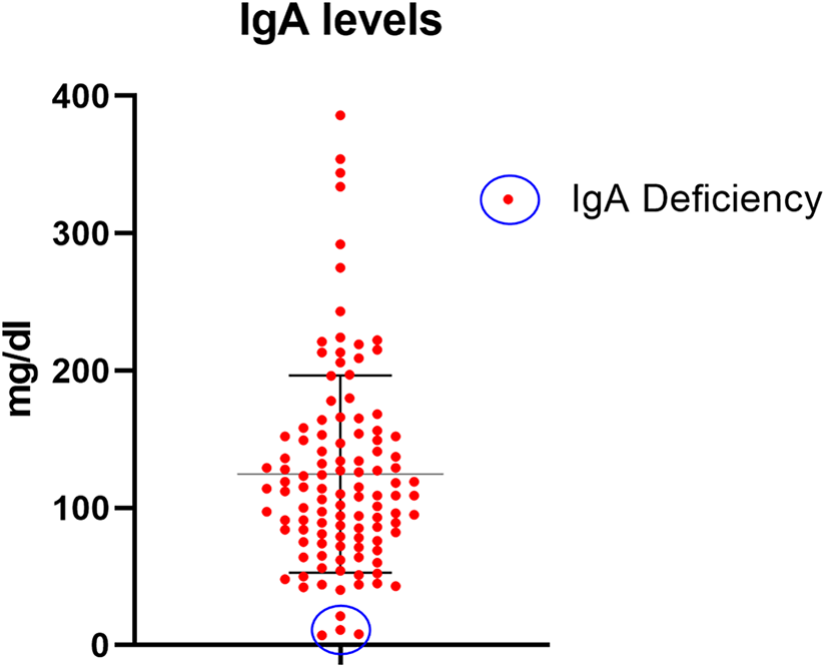
Total IgA levels for all studied patients with IgA deficiency cases (blue circle).

The tests performed and the sensitivity indexes of each test are presented in Table 4. The mean total IgA level was 123 ± 73 mg/dl. Four cases (3%) presented IgA deficiency and were diagnosed based on TGA and EMA IgG class tests (Figure 2).

**Table 4:** The tests performed and the sensitivity indexes of each test.

Methods	Positive	Negative
Genetic test DQ2+	93%	7%
Genetic test DQ8+	39%	61%
Anti-endomysium	80%	20%
Atrophy in biopsy (marsh III)	61%	39%

TGA IgA levels for non-IgA deficiency cases varied from 18 to 36,745U/ml with a non-normal distribution (Supplementary Figures 1A and B). The median TGA level was 138 U/ml with an interquartile range (IQR) between 67 and 988 U/ml. A logarithm base 10 transformation was performed using transglutaminase antibodies (TGA-L) (Supplementary Figures 2A and B). The TGA-L mean was 2.3 ± 0.8, and the TGA-L ≥1 represented the corresponding cut-off for positive TGA levels. The analysis regarding the age at diagnosis (<2 and > 2 years old), based on TGA-L, did not show a difference between groups (*p* = 0.09).

**Figure 3:**
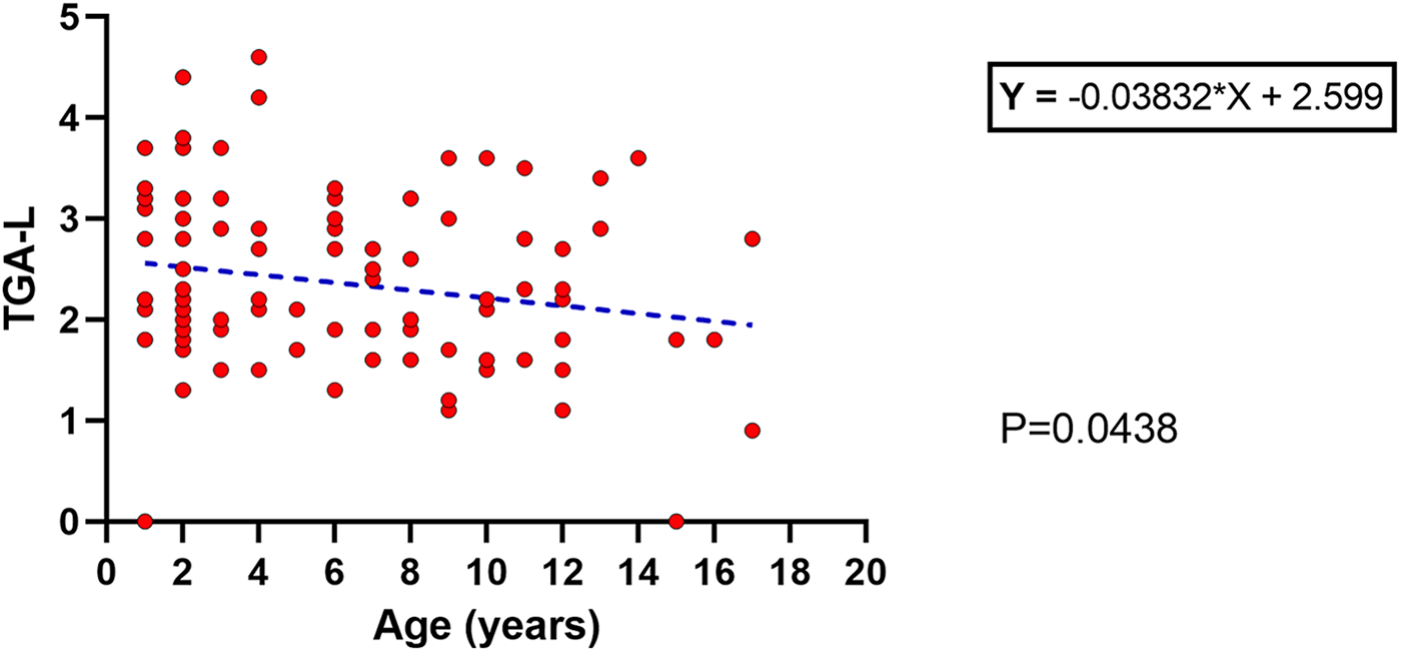
Linear regression with an inverse correlation between the age at diagnosis and the TGA-L; *p* = 0.04.

**Figure 4:**
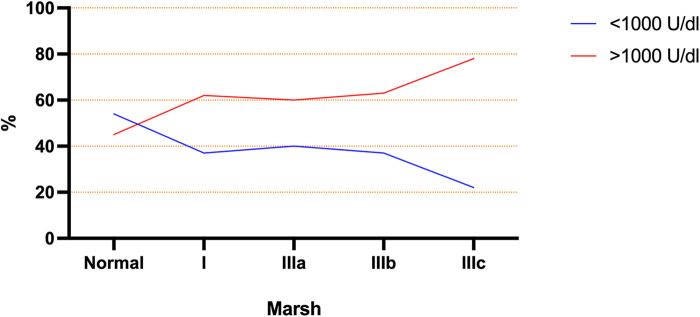
Modified marsh subtype groups represented by percentage of each group with TGA above and below 1,000 U/ml.

Based on the ESPGHAN criteria for CD (9), 63% presented TGA >10 times the upper limit. The mean age at diagnosis for this group (TGA >10×) was significantly lower than the group TGA <10 × (5.3 ± 4 vs. 7.4 ± 4 years old respectively; *p* = 0.017). Based on this finding, a linear regression model was performed and showed an inverse correlation between age and TGA-L levels, with a slow decrease over the years (*p* = 0.043) (Figure 3). Unexpectedly, 23% of the CD cases presented TGA levels higher than 1,000 U/ml (extremely high). The comparison between these TGA groups (>1,000 and <1,000 U/ml) showed a higher rate of atrophic profile in the first group (26% vs. 6.7%; *p* = 0.029). Additionally, the distribution of cases of TGA levels (<1,000 and >1,000 U/ml) among the Modified Marsh classification subtypes showed an opposite symmetric distribution between them (Figure 4).

### 3.5 Biopsy findings

**Figure 5:**
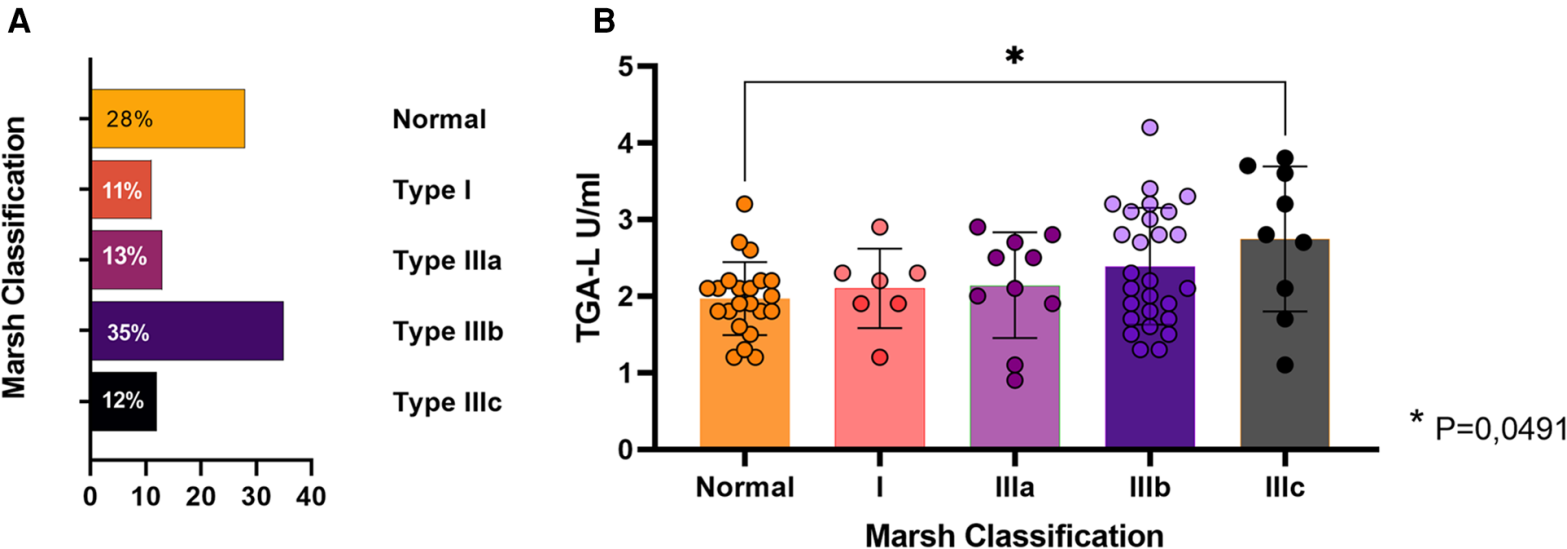
Duodenal biopsy findings. **(A)** Percentage of each Modified Marsh subtypes. **(B)** Average TGA-L by subtype. Presence of statistical significance between subtype 0 (normal) and IIIc; *p* = 0.04.

Overall, duodenal biopsy was performed at the diagnosis of CD in 76 cases (68%), and the most frequent subtype was type IIIb (35%) (Figure 5A). Among them, 31 cases (28%) presented non-atrophic profiles (Marsh 0 or 1) but TGA mean levels of 242.8 U/ml. As expected, the classical CD group presented a higher rate of atrophic biopsy than the Non-classical CD group (78% vs. 21%; *p* = 0.02). In general, a distinct transglutaminase antibody (TGA-L) was found between patients with atrophic vs. non-atrophic biopsy profiles (2.4 ± 0.8 and 2.1 ± 05 respectively; *p* = 0.022). In a sub-analysis including all subtypes of the Modified Marsh classification, a significant difference in TGA-L was identified solely between the normal biopsy and Type IIIb groups (adjusted *P*-value = 0.04) (Figure 5B).

Regarding the duodenal biopsy, a multivariate analysis showed a positive correlation for cases of atrophic biopsy profile and “growth deficit” (*T* = 0.218) and “TGA levels > 1,000 U/ml” (*T* = 0.033). A negative atrophic biopsy profile correlation was found for “constipation” (*T* = −0.277) (Table 5). All of correlations founded are classified as “moderate correlation” based on Kendall's correlation coefficient Strength (T).

**Table 5:** Biopsy profiles and correlations with clinical characteristics and tests performed.

Characteristics/tests	Non-atrophic	Atrophic	Correlation coefficient (*T*)	*p-*value
Gender female	37.2%	62.8%	0.053	0.65
Classical profile	30.8%	69.2%	−0.262	0.22
Age < 2 years old	37%	63%	−0.037	0.75
Constipation	64.7%	35.3%	−0.277	**0**.**015**
Growing failure	21.1%	78.9%	0.218	**0**.**05**
DQ2(+)	38.6%	61.4%	0.063	0.58
DQ8(+)	43.8%	56.3%	−0.090	0.51
TGA >1,000 (U/ml)	14.3%	85.7%	0.245	**0**.**033**
EMA (+)	41.5%	58.5%	−0.036	0.789

DQ2, haplotypes HLA-DQ2; DQ8, haplotypes HLA-DQ8; TGA, transglutaminase antibody IgA class; EMA, Endomysial antibodies IgA class; (T), Kendall's correlation coefficient.

*p*-value with statistical significance in bold.

## 4 Discussion

CD is an important systemic immune-mediated disorder triggered by the ingestion of gluten and occurs in individuals with a genetic predisposition. The reason for the onset has a complex immunogenetic mechanism that has not been completely elucidated until now (15). Transglutaminase is the most important factor in its pathogenesis and its main action is to deaminate specific gliadin fragments in the lamina propria, binding them to HLA molecules expressed on the surface of antigen-presenting cells (6). The process continues with the HLA-DQ system, which presents gluten epitopes (highly antigenic) to CD4 + cells and is responsible for the immunologic response to antibodies against tissue transglutaminase, gliadin, and endomysium (8).

This important pathogenesis factor (transglutaminase) also represents the most important serological diagnostic test for CD. It has a sensitivity of up to 90% and a specificity of approximately 96% (16, 17). Catassi et al. identified a sensitivity for TGA of 0.93 (95% CI of 0.88–0.97) which was the same sensitivity identified in our study (0.93) (18).

Interestingly, most cases (63%) had levels of TGA 10× upper limits in this study. A subgroup (23%) had levels above 1,000 U/ml (55 times the upper limits). In general, the TGA analysis is carried out up to 200 U/ml which is sufficient to achieve greater than 10 times the upper limit of the normal range. Although it can be detected up to 4,000 U/ml by some commercial kits (19). In this study, the TGA levels carried out to maximum possible dilution (titration), and it was the motive to find some cases with extreme levels of TGA. The reason for extreme TGA levels is unclear. This finding to be related to a high gluten consumption at time of diagnosis or maybe it represents a distinct immune response for some cases (1, 2). Additionally, it is unknown whether these cases have distinct manifestations of CD. In our analysis, cases with extremely high TGA levels presented a moderate positive correlation with the atrophic biopsy profile (four times more atrophy than the group with TGA <1,000 U/ml). This issue has not been discussed in the literature, but it is a source for new studies to determine the possible reasons for higher transglutaminase levels and whether it represents specific CD findings.

Our study identified TGA levels ranged from 18 to 36,754 U/ml with a non-normal distribution. Owing to these large variations, the median TGA was not used in the analysis because it represents an inexact variable. While the mean is the average of all the data values, the median is the half position of the values. Thus, we used a statistical approach named “Log transformation in base 10”, which is used for data with large variation, such as the TGA levels. Logarithmic transformation is a statistical tool that assumes constant variance in the context of linear modelling with highly reliable results. Smarrazzo et al., in a study of CD diagnosis methods, across mediterranean countries, used a log transformation for TGA levels to analyze sample sizes with non-normal distribution (14). Based on this study, we used the same statistical strategy for transforming the TGA levels to obtain a normal distribution. This allowed us to compare the mean TGA with other important variables (clinical manifestations, other serological tests, and histological findings) and to correlate the TGA-L with other continuous variables, such as age at diagnosis, preserving the reliability of the results. So, whether TGA levels show great variability with a non-normal distribution in a population studied, logarithmic transformation is a statistical resource that should be considered.

Classical CD cases present important severity for symptoms and have been related to higher intestinal atrophy (20). In our study, we identified the classical CD group presented a higher rate of atrophic biopsy than the Non-classical CD group as well (*p* = 0.02) which has been identified by other studies (20).

An important finding was the significant rate of duodenal biopsies performed at the time of diagnosis (64%). The rate of biopsies performed according to the ESPGHAN criteria for CD diagnosis is currently decreasing. We attribute this high rate of biopsies to the considerable number of cases of non-classical CD with recurrent abdominal pain as the main symptom. This has motivated the inclusion of endoscopy with duodenal biopsy for diagnostic evaluation. A cross-sectional CD screening population study in Sweden (2014) analyzed 13,279 children who were tested for TGA, and positive cases were selected for small intestinal biopsies. It showed that the biopsies could have been omitted in only one-fourth of all of CD cases (21). In our study, the rate of Mash 0 or I represented 39% of cases who performed duodenal biopsies (43 cases). These cases were included in this study because they presented criteria for CD diagnosis according to ESPGHAN. However, they did not present an atrophic biopsy profile. This subgroup analysis showed a mean TGA equal to 242.8 mg/dl, and half of these cases were classified as classical CD. It is known that a considered rate of cases can present with transglutaminase levels >10 times, but no important biopsy abnormalities.

TGA was correlated with aging and the degree of intestinal damage in this study. The first correlation (TGA and aging) has been reported in a contradictory statement. In the early 2000s, Baldas et al. identified an increase in TGA with increasing age in CD patients (22). In contrast, Vivas et al. (2008) found that TGA is inversely correlated with age (23). Another study published in 2020 found no difference in TGA levels among age groups (24). In this study, we identified a decrease in TGA levels related to the aging of cases at diagnosis. For that analysis we used a linear regression model, which has better accuracy than the one used in the aforementioned studies (average or median comparisons).

The second correlation identified in our study was between TGA and the degree of intestinal damage. There is a significant correlation between TGA titers and severity of duodenal histopathology according to the literature (25). We identified a positive correlation which the increase in the mean TGA-L was related to the intensity of intestinal damage. Based on an analysis of the subtypes of the Modified Marsh classification, a significant difference was reached between the normal biopsy and type IIIc groups. This finding has also been identified by other authors (16, 17, 26).

HLA-DQ2 and HLA-DQ8 are the main haplotypes related with genetic markers of CD. Almost 95% of CD cases express HLA- DQ2 or HLA-DQ8 (27) but authors have identified this condition at distinct rates (28–30). According to the ESPGHAN updated guidelines for CD published in 2022, genetic marker tests are not recommended for all suspension cases of CD. Only in cases of uncertain CD diagnosis before gluten exposure (31). Our study was a retrospective cohort study, and the genetic markers were considered in the analysis for all cases because they were performed frequently in the last decade.

In 2017, Basturk et al. identified 24% of HLA-DQ2 and DQ8 negative tests. They did not show any significant difference between the negative genetic markers group and the DQ2/DQ8 positive cases regarding CD manifestations and diagnostic tests (29). We identified both negative genetic marker expressions in 4.5% of CD cases for DQ2.2, DQ2.5 and DQ8 (Olerup SSP® HLA typing kit) and these cases did not present differences in all parameters and variables analyzed (clinical manifestation, tests performed, and biopsy findings). Genetic markers are crucial to the development of CD, but some cases can have the absence of the main genetic markers (29). In this study, the DQ2.2, DQ2.5 and DQ8 were performed but other markers can be found in CD patients (4). It is important to emphasize that we identified cases with negative genetic tests however they may be positive for other genetic tests not performed in our study (a false negative). Regarding these specific tests performed (DQ2.2, DQ2.5 and DQ8), Immunogenetic lab at the hospital has performed DNA amplification as a double test for both positive and negative cases.

The genetic haplotypes were inherited from the patient's parents. It is estimated approximately 40% of the general population have these haplotypes (HLA-DQ2/DQ8); however, only 1% of them will develop this disease (1, 2). In this study, we identified the presence of haplotype HLA-DQ2 in 35% of First-degree relatives. Only 15% of families had confirmed CD among parents or siblings. Tolene at al. identified a family history of CD in 19% and not significantly associated with any of the clinical and demographical data analyzed or the belonging to a certain HLA-DQ class risk (32). Genetic variability is constant in families and cannot be predicted in the development of CD among FDRs. Mansouri et al. identified a rate of 44% of DQ2 positivity among 100 FDRs of CD, which is close to that found in our study. This study concluded that HLA typing is not effective in predicting CD among FDRs of CD patients (5). An interesting finding in our study was that the father was markedly the main FDR with a significant positive genetic test rate (69%) compared to other First-degree relatives. It is an intriguing question whether the disease is more common in women (>60%); why is the father the family member with the highest prevalence of the DQ2 positive? A retrospective observational Spanish study (2019) emphasized that patients with a family history of CD should be investigated for CD when HLA-DQ test is positive (33). A better understanding of the hereditary inheritance of the HLA system in families with CD is an important topic to address in future studies.

The HLA-DQ2/DQ8 genotype expression is considered a high-risk factor for the early onset of Type 1 diabetes as a “genetic accelerator” (34). A study in European Caucasians showed that HLA-DQ2 is a positive risk factor for type 1 diabetes however HLA-DQ8 (DQB1* 0302) is the main genetic marker of greater susceptibility to diabetes in children (34–36). In our study, type 1 diabetes was found in 5.8% and these cases showed a significant DQ8 expression however, they represented a small number of diabetes cases (6 children) and does not allow a general conclusion to be drawn. The diabetes rate estimated in CD population is 2%, according to the populational study including United States, Finland, Germany, and Sweden (37). The literature emphasizes a strong relationship between HLA-DQ8 and diabetes. Surprisingly, when we compared the DQ2 positive and negative groups, the DQ2(−) group had a higher rate of patients with diabetes than the DQ2(+) group (25% vs. 4%) with significance. The presence of DQ2 may be crucial for the development of CD but is not crucial for the development of other autoimmune diseases such as type 1 diabetes (37). Although, when the diabetes population without CD is analyzed, an important expression of DQ2 can be observed (28).

The limitation of this study is due to the design (a retrospective cohort study), in which the accuracy of the information reported cannot be controlled. A potential bias of the study is that the data came from a single center representing a specific and restricted population, which corresponds to the small sample size of pediatric CD population. Additionally, DQ8 was not tested in 27% of the population which can have influenced the results of negative genetic markers rate.

## 5 Conclusion

Regarding diagnosis tests, the results of this study seem to indicate transglutaminase levels decreased with the age of the pediatric population at diagnosis time. Transglutaminase presented a moderate positive correlation with intestinal damage on biopsy. Cases with atrophic biopsy profile had moderate positive correlation with growth retardation and extremely high transaminase levels. Furthermore, the atrophic biopsy profile showed a moderate negative correlation with constipation.

In terms of genetic markers, 93% were HLA-DQ2 positive. Negative genetic markers (HLA-DQ2/DQ8) represented a small number (4.5%). DC cases with Type I diabetes presented a significant rate of DQ2 negative and DQ8 positive. The positive DQ2 haplotype was significantly higher in cases with a family history of CD. The father was the most common positive relative with this genetic marker among first-degree relatives, DQ2(35%).

## Data Availability

The raw data supporting the conclusions of this article will be made available by the authors, without undue reservation.
