# Supplementary material for: Celiac disease diagnosis: transglutaminase, duodenal biopsy and genetic tests correlations
Source: Front Pediatr. 2024 Aug 29;12:1330511. doi: 10.3389/fped.2024.1330511 (PMC11390444; doi:10.3389/fped.2024.1330511)
Supplement: Supplementary file 1 [file Datasheet1.pdf]

# Supplementary figures:

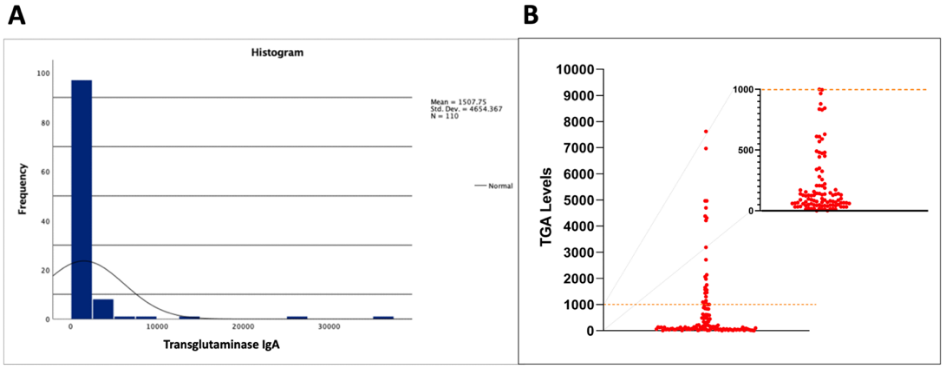


Supplementary Figure 1

Transglutaminase levels of cases. (S1A) Histogram and (S1B) scatter plot of in patients with a non-normal distribution.

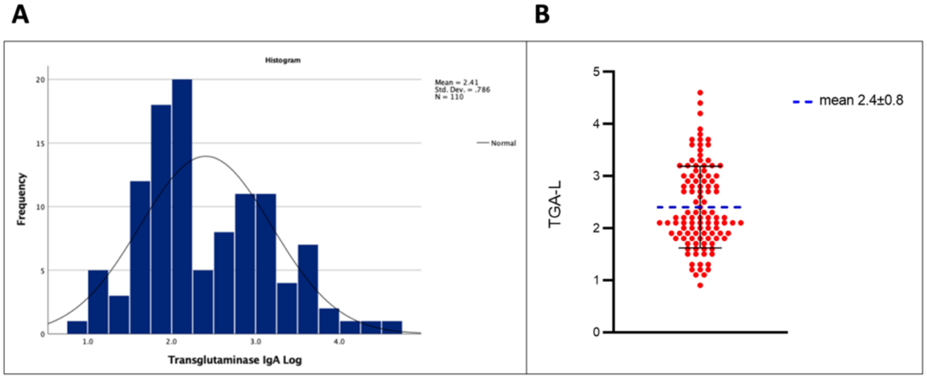


Supplementary Figure 2

Transglutaminase levels of cases. (A) Histogram and (B) scatter plot after logarithmic transformation (TGA-L) with a normal distribution.
